# Hardware implementation of visible light communication based multipurpose camouflage spy robot

**DOI:** 10.1038/s41598-025-89134-6

**Published:** 2025-03-04

**Authors:** R. G. Sangeetha, S. Krithika, C. Hemanth

**Affiliations:** School of Electronics Engineering, Vellore Institute of Technology-Chennai, Chennai, Tamil Nadu 600127 India

**Keywords:** Visible light communication, Spy robot, Little’s theorem, Graphical user interface, Engineering, Electrical and electronic engineering

## Abstract

The proposed system outlines a novel camouflage-enabled mobile robot designed for various roles that require secure and rapid audio, video, and navigation data transmission and the capabilities to camouflage with any background to aid in remote monitoring. The robot is equipped with a self-sustaining power management and obstacle detection system. The design addresses the challenges of converting operations by integrating state-of-the-art Visible Light Communication Technology for secure and rapid communication. The system’s performance is analyzed using statistical methods and Little’s theorem, providing insights into operational efficiency and transmission reliability.

## Introduction

Visible Light Communication (VLC), a subset of Free Space Optical Communication, uses the visible light spectrum (400–800 THz) as a medium to communicate or transfer information. VLC has gained attention in recent years as a reliable and secure alternative to Radio Frequency (RF) communication in applications requiring low electromagnetic interference, larger bandwidths, and higher data rates^1^. However, VLC faces some challenges such as the limited range of light and sensitivity of VLC systems to ambient light^2^.

Since VLC works on a line-of-sight basis, light signals are resilient to obstructions like walls^3^. This feature ensures higher security since unauthorized users cannot intercept signals outside the designated area. Since light travels faster than radio waves, data transfer in VLC systems can occur almost instantly. Compared to RF systems, which frequently need substantial infrastructure for antenna and signal boosters, VLC systems can be simpler and easier to install.

The main components of the VLC system are the transmitter and receiver. The transmitter consists of an LED and driver circuit. The driver circuit controls the LED’s modulation, which transforms digital data into the proper light signals^4^. It guarantees that the LED functions efficiently for data transfer. The receiver typically consists of photodetectors, optical filters, and amplifiers. Photodetectors^5^ convert the modulated light signals to electrical signals, which is passed through the amplifier to increase the signal levels. Optical filters eliminate interference from ambient light and other undesired sources.

In the field of robotics, robots can efficiently and rapidly transfer data from a variety of sensors by using VLC^6^. Large data transfers, like video feeds from cameras or telemetry from sensors, are made possible by VLC’s high bandwidth, which is crucial for applications requiring real-time analysis and decision-making. VLC can let machines share safety-related data, such as speed and braking status, in situations like industrial robots^7^ or driverless cars. This real-time communication can enhance operational safety and help avoid mishaps.

### Use case and industries

The various applications where VLC-based camouflage spy robots can be used are discussed below.

1. *Military and defense* Camouflage robots can be deployed for border patrolling or for risky missions that would pose a great threat to the lives of military personnel. Camouflage robots were designed to blend into the environment and perform operations without being noticed easily. In environments that require discreet communication, VLC^8^ can be used with camouflage techniques to reduce detection while maintaining robot functionality because it uses visible light. The robot can function in settings where signal emissions from conventional RF communication might reveal its location.
2. *Environmental monitoring* Camouflage robots can be deployed to assess and learn about the wildlife in their natural habitat^9^. Unlike static animal dummies with fixed viewpoints, these robots can move freely, capturing dynamic footage and data vital for research and documentation. National Geographic channel uses animal-like dummies by replacing the eyes with a camera for documentation. However, such dolls are static and cannot move around; this invention can also be used for such use cases.
3. *Private security firms* This robot can be deployed in areas that require high security providing advanced monitoring capabilities without drawing attention.
4. *Search and rescue operations* In disaster scenarios^10^ such as earthquakes, the camouflage robot can help in search and rescue operations by navigating risky environments, locating survivors, and using its camouflage feature to approach survivors without alarming them.
5. *Mining and exploration* in hazardous mine^11^ areas, with toxic gases which when inhaled by humans for a longer period of time might cause incurable ailments. In such regions to monitor and inspect the areas, the proposed camouflage robot could be used to enhance safety and efficiency in underground operations.

### Related works

The most important components in any VLC system are the light source (transmitter) and the receiver. Though the most optimal choice is Light-Emitting Diodes (LEDs), since the camouflage robot would be deployed outdoors, the literature^12^ suggests using laser as the ideal source of light for the application of the camouflage robot. The work^12^ analyses various factors such as the Confinement Stark Effect (QCSE), crystal orientation, carrier lifetime, and recombination factor, along with the performance of the VLC system.

Point size receivers are impractical due to the camouflage robot’s mobility and distance from the receiving station, and natural interference must be considered. Using solar panels to detect laser lights in a VLC system was left as an open topic for future research by the authors in^13^. The camouflage robot presented in the current work investigated this issue. The ability of solar panels to convert modulated light signals into electrical signals without the need for external equipment is well known. Additionally, as stated in^14^, which discusses the dual use of solar panels for energy harvesting and as a receiver in VLC systems, the DC component of the modulated light can be harvested in the receiver. The current study’s motivation and uniqueness is to combine the laser and solar panels in an outdoor mobile system.

VLC is less vulnerable compared to other communications methods in terms of security. To further minimize the vulnerabilities, the work in^15^ summarizes all security techniques proposed in the literature on VLC systems so far. The literature^15^ addresses physical layer security, transmission jamming, possible data modification, secure localization, key generation, steganography, and VLC in vehicular networks.

Simultaneous real-time audio/video transmission has its challenges. The work in^16^ explores the experimental performance of audio transmission using VLC and achieves up to 4 Mb/s data using OOK modulation. It also provides step-by-step guidance for setting up a VLC system for data transmission. The work in^17^ describes video and audio transmission using VLC indoors. The intensity of audio and video received with respect to the distance between the source and receiver is observed and analyzed.

VLC has been extensively used for real-time localization and positioning. Using visible light positioning, the research^18^ introduces a novel high-precision indoor localization method for mobile robots that can support up to 20 km/h and achieve a positioning accuracy of 3.231 cm. This system shows great promise for low-cost positioning solutions in various applications by using smart LED lighting with VLC and Bluetooth control. A prototype to transmit alphanumeric or picture data was created in^19^ for industrial applications to promote efficient industrial automation, enhance real-time monitoring, communication, safety and security. The article^6^ discusses the integration of modern localization technologies in the context of Industry 4.0, with a focus on 5G and Visible Light Communication (VLC) technologies. It highlights preliminary measurements indicating that the communication system can reliably operate over distances of up to 45 meters when the receiver is in line of sight (LoS) with the transmitter. The Wheeled Robot Chain Control System proposed in^20^ aims to enhance the inspection of underground facilities by using VLC to address the limitations associated with current mobile inspection systems. These systems often face challenges such as limited inspection distances due to unstable wireless communication and cable restrictions in complex environments.

The War Field Spy Robot proposed by authors in^21^ is a military tool with motion sensing for live streaming, a 360-degree night vision camera, and metal detection technology to identify metallic objects in combat areas. The study in^22^ describes a complex model of an intelligent spy robot equipped with cutting-edge surveillance capabilities. It has a high-definition camera that broadcasts live footage through a Python server and a 2.4 GHz bi-directional wireless communication device. A cloud server houses the data that the robot collects, making management and access simple. To broadcast the live streaming, they required Wi-Fi^21,22^, which could be challenging to get in actual combat zones. Furthermore, they haven’t adapted any camouflage techniques.

The literature^23^ provides a brief overview of VLC technology for outdoor navigation along with some emerging areas of application for VLC-based outdoor positioning systems. The complex relationship between outdoor VLC systems and ambient light for navigation is explored in^24^. This article includes the calculation of angular error for the angle of arrival and the measure of received energy across sensors to assess ambient light’s influence on system performance. It also facilitates a detailed analysis of sunlight effects which would help in optimizing the positioning of the transmitter and receiver. This would greatly improve the design of the camouflage robot by adding the feature of dynamically adjusting the solar panel to the most optimal position with respect to the static station at all times.

Using integrated color sensors positioned thoughtfully throughout the robot’s body, the camouflage robot system designed in^25^ is made to recognize the colors of its surroundings. The robot’s onboard processing unit receives information from these sensors about the colors of the near surroundings. To replicate the identified colors in real-time, the system subsequently creates matching color patterns that are shown on an LED matrix. The ability of this technique to do covert monitoring is hampered by a few issues. The performance of the colour sensors used may limit the accuracy of colour detection and the display on the LED matrix. The effectiveness of camouflage may be compromised if these sensors are unable to reliably detect the actual colours of the environment, leading to irregularities in the colour patterns that are presented. While various image processing techniques could be used to accomplish the camouflage feature of the robot,^26^ uses image merging concept where the images of the surroundings are captured and are merged to form an image which is displayed on the screen that covers the body of the robot. They displayed the images using LCD, which has a lower resolution and refresh rates. A soft robot capable of adaptive camouflage using thermochromic materials is described in^27^. This system utilizes advanced pattern generation to mimic natural environments in real-time, overcoming traditional limitations of camouflage, such as lateral pixelated schemes. By integrating an active control system and sensing units, it retrieves the background color and instantaneously matches its surface.

The proposed system extends the scope of VLC in robotics by integrating multimedia transmission, environmental adaptability, and sustainability into a single platform. It advances the field by demonstrating a practical, multifunctional robot capable of real-time communication and autonomous operation in military and covert applications. This project sets the stage for future research exploring high-speed, secure, and energy-efficient VLC applications in robotics.

The main contributions of the work are:

- We use VLC to transmit audio, video, and navigation in a single system, which is a step forward in robotics. Incorporating solar panels as a receiver doubles as a power source, addressing communication and energy efficiency challenges. Previous studies have demonstrated using VLC in robotics primarily for one-way data transmission or simple signaling like navigation guidance and data streaming.
- We integrate advanced VLC communication systems with camouflage. Few research projects have explored active camouflage in robotics, often restricted to static pattern adaptations or low-resolution approaches.
- Several projects focus on solar-powered robots or hybrid systems, but we simultaneously exploit the solar panel for both power generation and communication.

The rest of the manuscript is organized as follows: “System design & methodology” section elaborates on the system design and methodology, highlighting each individual component of the system. “Observation” & “Results” sections present the observations and results, respectively. Finally, conclusions, along with limitations and possible future improvements, are presented in “Conclusion and future scope” section.

## System design & methodology

The integration of multiple systems adds complexity, and this complexity is reduced by following a modular approach where each module of the system is developed and tested independently before being integrated. This allows each module to function as a distinct module making it easier in troubleshooting. Different levels of modularity of this system is explained ahead.

The design of the system is mainly classified into two main modules, namely, the mobile robot (transmitter) and the static station (receiver). Since the system uses duplex communication, that is, the navigation commands for the robot is sent from the static station and the video and audio streams captured by the mobile robot is transmitted to the station where it is processed and displayed on the user interface. For easy understanding, the transmitter will be addressed as the mobile robot and the static station as the receiver throughout this paper.

**Fig. 1:**
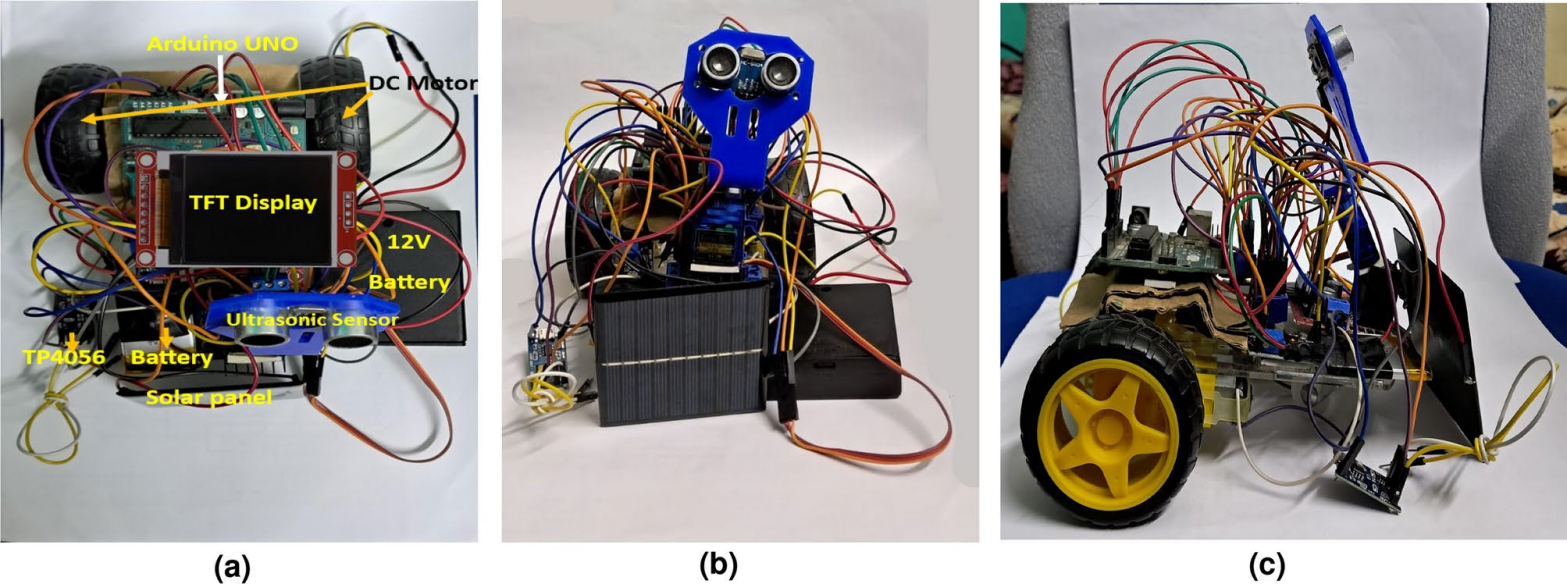
Mobile robot.

The mobile robot consists of an Arduino UNO that controls the motors to move as commanded by the user through the user interface, an ultrasonic sensor to detect and avoid obstacles, a solar panel to receive navigation commands and also to charge the onboard battery that powers the Arduino UNO. The surveillance unit consists of an ESP32 CAM module attached to the INMP441 MEMS mic and a laser, to capture and transmit real-time video and audio streams respectively. In order to merge the robot seamlessly with its surroundings, an ESP32 CAM module attached with a 1.8-inch TFT display was used. The top, front and side views of the mobile robot is shown in Fig. 1a, b and c

### Surveillance unit

**Fig. 2:**
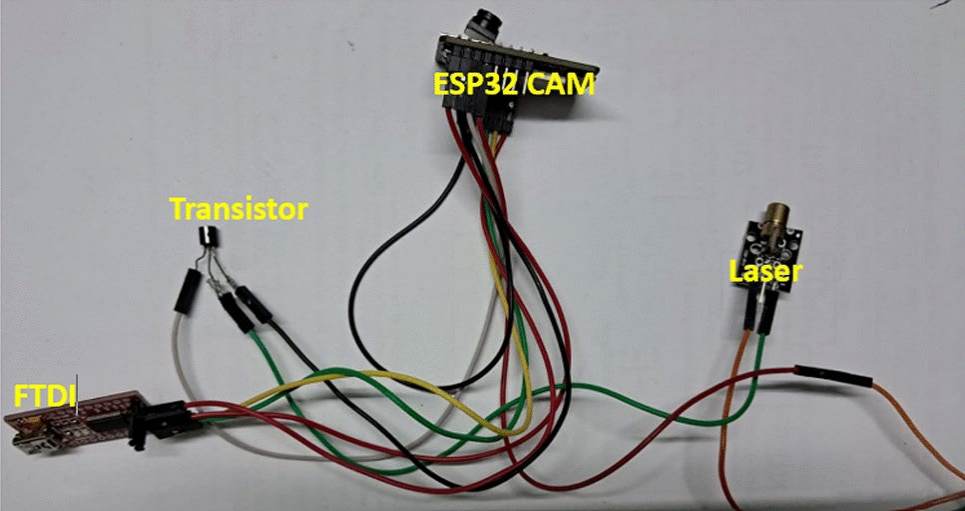
Surveillance unit circuit.

This unit consists of an ESP32 camera module connected to a mic, laser. Since the laser’s working voltage is 5V and ESP32 works on 3.3V logic, to amplify the voltage a transistor is used as shown in Fig.2. Since VLC requires a line of sight for communication the camera mounted on the mobile robot, on startup, takes a snapshot of the environment and checks the feasibility of VLC in the region. As visibility is important for effective communication, the snapshot taken is processed using computer vision techniques to analyse if the visibility is higher than the threshold required for effective VLC communication. If the visibility is acceptable, the robot is enabled and functions normally, else the message that VLC is not feasible is displayed and all functions of the robot are disabled.

In regions where VLC is feasible, navigation of the robot is remotely controlled. The camera module and mic are connected to two different GPIO ports. The firmware on the microcontroller creates tasks to read data from these ports simultaneously. The opensource FreeRTOS is used for this purpose. These feeds are converted to binary by the microcontroller and transmitted using the laser. This transmission is received by the solar panel at the static receiver end which in turn is connected to a computer.

The audio is transmitted at all times from the robot to the receiver station. The user has the control to choose to stream video when audio is insufficient and video is required. The audio and video feeds are captured and sent to the receiver station using VLC through the attached laser on its body. The frames are received and converted to audio files and images. The frames of images are then displayed on the interface creating a video with the audio file playing in the background.

### User interface and receiver

**Fig. 3:**
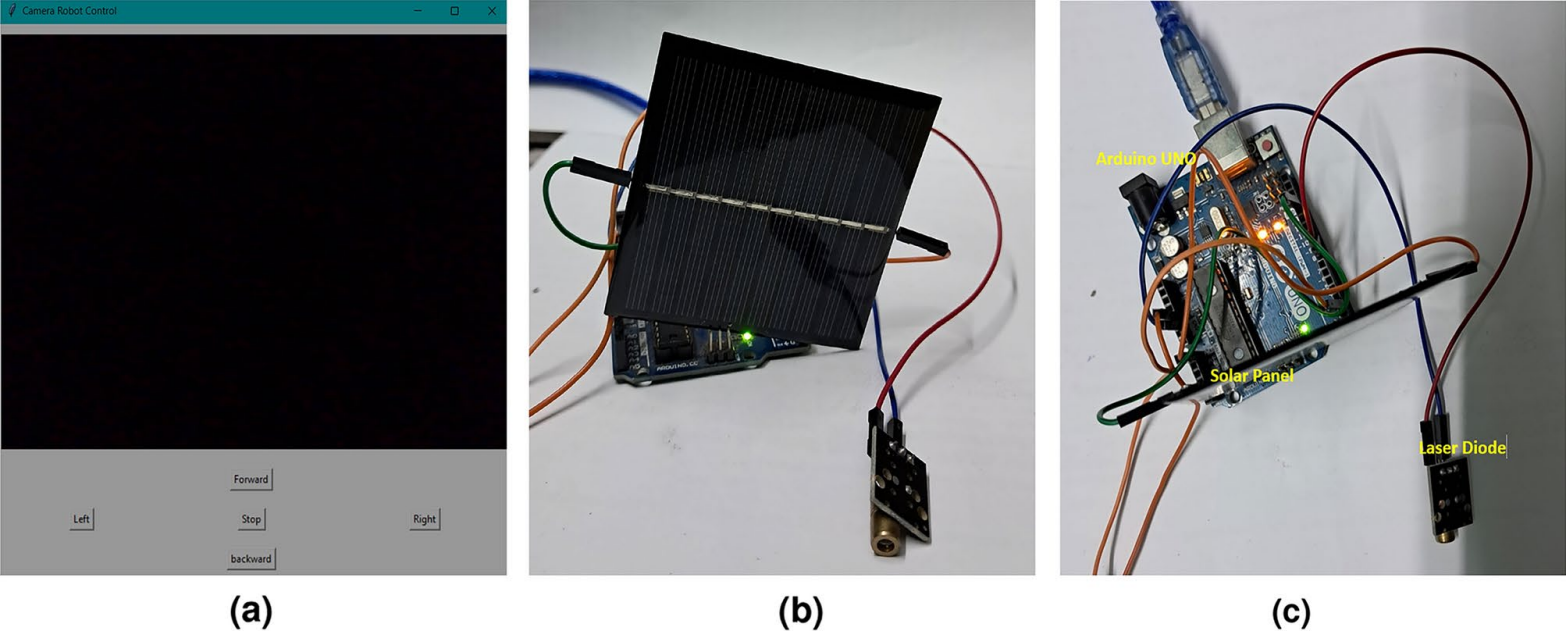
GUI and receiver.

The receiver has a simple hardware setup - a microcontroller, a solar panel (that also acts receives audio/video streamed from the robot), and a laser for transmitting navigation commands. This hardware setup is interfaced to a GUI, created using Tkinter. The GUI is to send navigation commands to the mobile robot and to receive and play real-time audio and video streams. The Tkinter app (shown in Fig.3a), has navigation buttons to control the robot remotely and to play the audio/video received from the robot.

Each navigation direction button in the GUI generates a code that is communicated using serial communication to the hardware setup. When a button is pressed, the corresponding direction code is generated and transmitted to the mobile robot, as a series of binary codes using a laser diode. Each direction code is a data frame of 6 bits. The first bit is the start bit and the next five bits uniquely represent the direction. The direction codes have been selected in a way in which, even when a bit gets lost, a code of a different direction is not represented thus, enabling accurate navigation. On-Off Keying mechanism is used during transmission. The mobile robot receives these unique codes, decodes them and moves accordingly to the direction identified. The GUI interface is shown in Fig. 3a, and the front and top views of the receiver module are shown in Fig. 3b and c.

### Navigation unit

**Fig. 4:**
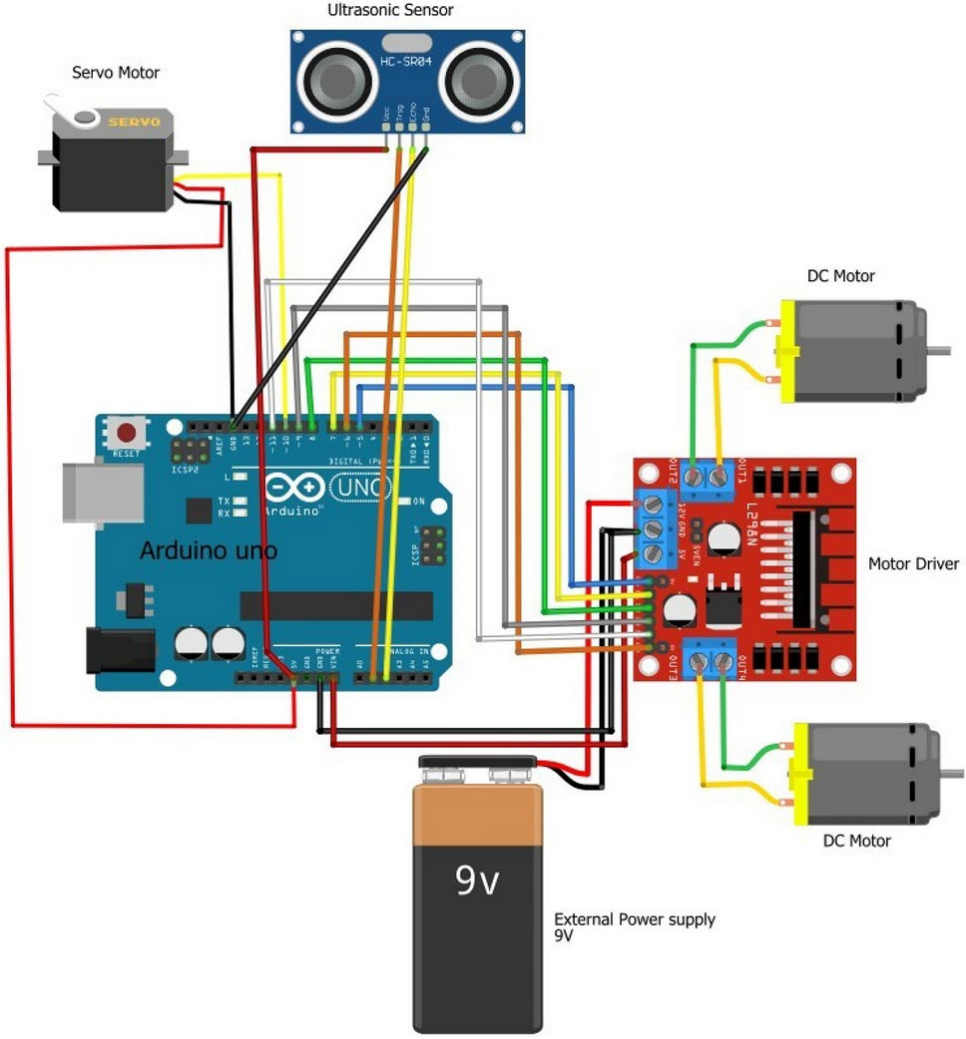
Navigation circuit.

The data frame sent by the receiver is captured by the solar panel connected to a microcontroller. The data frame thus received is decoded and the direction identified. Using this the controller signals the motor driver connected with it to switch the motors on or off and also defines the speed. This is termed as ‘manual’ mode of operating the mobile robot. It is possible to set an ‘automatic’ mode. In this mode the navigation is done autonomously by detecting and avoiding obstacles using the ultrasonic sensor connected to the motor control unit. The circuit diagram of the navigation unit is represented in Fig. 4

### Battery management unit

All mobility robots are battery-operated. This means that they have be connected to a charger station for some period of time in-spite of modern technologies being deployed to reduce their power consumption and increase efficiency. During this time period required for charging, the mobile robot would be inactive. This work proposes a dual role for the solar panel: (i) as a receiver of visible light communications (VLC); and (ii) as a battery charger.

**Fig. 5:**
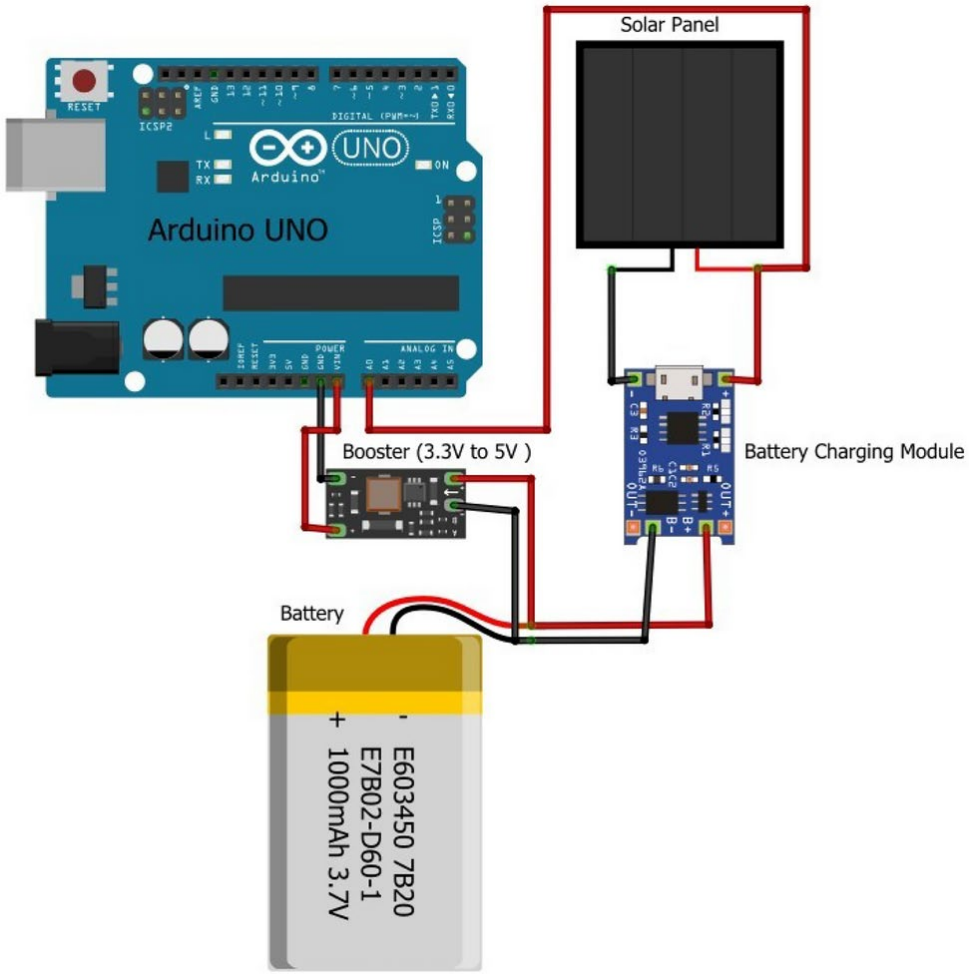
Battery management circuit.

The battery management unit is coupled with a TP4056 battery charger module and booster, to ensure effective power for the microcontroller/motor controller. In this prototype, the maximum voltage generated by the solar panel is 6V. Hence a 3.7V LiPo battery is used to power the microcontroller. However, higher-voltage solar panels can be used to charge the motor batteries and power the microcontroller. The battery charger module (TP4056) is a complete constant current/constant voltage linear charger for single-cell Li-ion batteries with a discharge protection module. This provides a 1A charging current and it cuts off when charging is complete. When the battery voltage drops below 2.4V, the load is disconnected and protects the cell from operating at very low voltage. Protection against over-voltage and reverse polarity is provided. The TP4056 also stabilizes the current generated the solar panel which changes constantly. This IC provides reliable charging by providing overload and short circuit protection. The voltage of the solar panel (6V), can charge a 3.7V battery. As most microcontrollers work with 5V, an amplifier module is provided to boost the 3.7V to 5V. The circuit diagram of the battery management unit is as shown in Fig. 5.

#### Power generation using solar panels

The proposed system uses solar panels as a receiver and battery charger. The prototype designed in this work uses a 6 V, 100 mAh solar panel with a 6 cm $\times$ 6 cm dimension.

1 $$\begin{aligned} Energy (Wh) =Voltage (V) \times Current (Ah) \end{aligned}$$

Using the above equation, the energy generated can be calculated as 0.6Wh. This energy generated using solar panels complements the energy supplied by the battery to the system. If the size of the solar panel is increased, energy conservation will also increase. For example, if a 6 v, 180 mAh solar panel of 9.9 cm $\times$ 6.9 cm is used, the energy generated would be 1.08 Wh. Applications requiring high power would benefit from the proposed system.

### Camouflage unit

**Fig. 6:**
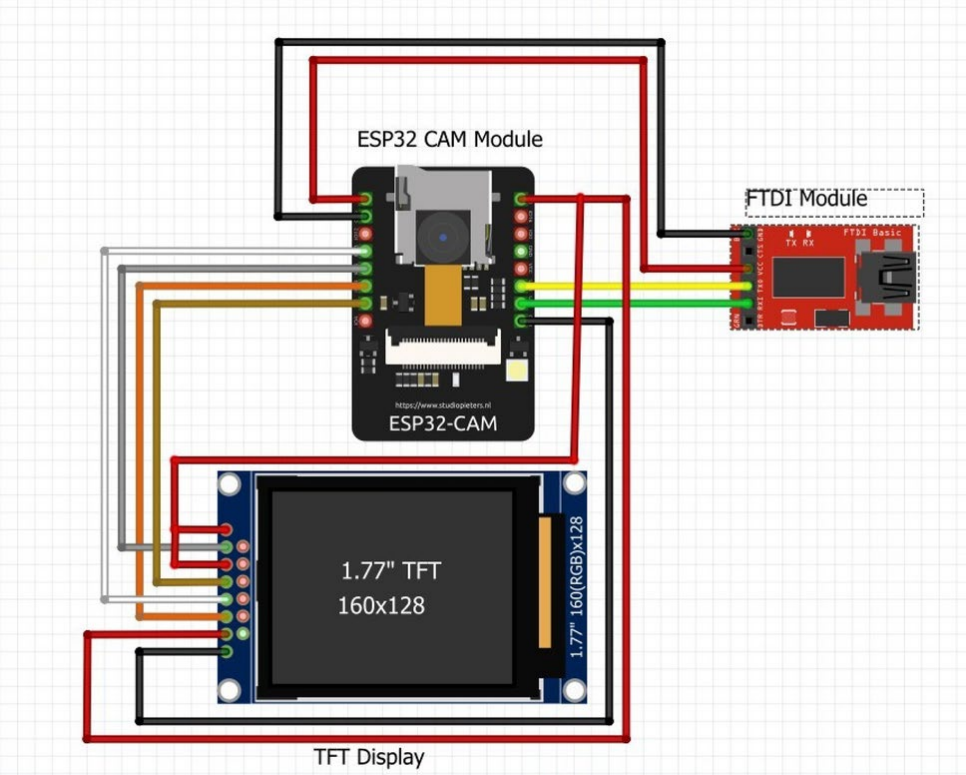
Camouflage Feature.

This work proposes incognito spying operations using a camouflage feature to merge with any environment in which it may be deployed. A microcontroller attached to the camera and TFT display(s) are used to accomplish this. This camera module is placed facing the ground underneath the robot chassis to capture the ground patterns effectively. The captured images are displayed on the TFT display, which envelopes the robot’s body, making the robot indistinguishable from its environment. The circuit diagram is as shown in Fig. 6.

### Hardware testbed

**Fig. 7:**
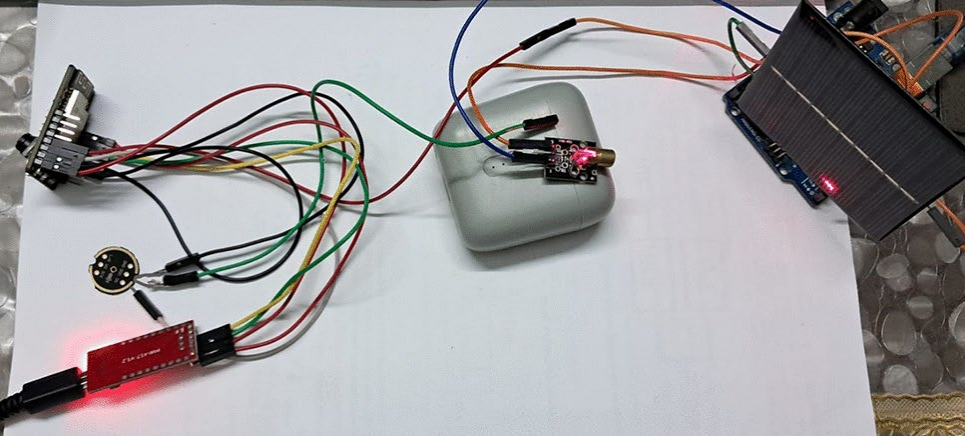
Audio and video transmission setup.

Figure 7 represents the experimental setup of the surveillance unit which was later loaded on the robot’s chassis. Capturing and streaming the image feed was done in different lighting conditions, and it was observed that the buffer length, that is, the length of a picture data frame, was directly proportional to the level of illumination and was also directly proportional to the number of objects that were captured. Similarly, the audio feed’s buffer length, or the length of an audio data frame, was directly proportional to the audio frequency. The relation between the buffer length and time is taken for each audio/video frame to be transmitted and received was observed and analyzed. The transmitter and the receiver were 40 cm apart for all the readings.

## Observation

The Visible Light Communication system employed in this setup uses the On-Off Keying mechanism. The variables considered in the experiment are buffer length, illumination, arrival time, and departure time of the image. The buffer length, or the amount of data awaiting transmission or reception, fluctuated based on illumination levels and the number of objects detected in each image. Higher illumination correlated with an increase in buffer length, which, combined with more detected objects, led to extended transmission times. Arrival time is the time at which the audio/video data reaches the buffer and departure time is the time at which the last data bit is transmitted and the buffer gets filled with the next set of image data.

The buffer length of each video and audio stream along with its transmission time was observed to analyze the system’s performance and is tabulated in Table 1. Looking at the average transmission time with the change in buffer length provides insights into the transmission load and complexity. Calculating standard deviation measures how the transmission time is spread out around the mean, suggesting the variability or inconsistency in transmission times that could impact system performance. The Pearson correlation coefficient is also calculated to understand the relationship between buffer length and transmission time.

Furthermore, to analyze the characteristics of this VLC system, Little’s theorem from queueing theory is used. Another set of dataset was created for this purpose by observing the VLC system and is tabulated in Tables 2 and 3.

**Table 1:** Buffer length with corresponding transmission time.

Buffer length		Transmission time in seconds	
Video	Audio	Video	Audio
122944	4062	11.569	0.6982
159760	5553	14.752	0.3652
224936	6211	20.419	0.3649
270912	6217	24.343	0.6797
305928	6273	27.433	0.6736
351728	6677	31.445	0.3655
393792	6685	35.041	0.3719
441128	7357	39.164	0.3901
471984	8343	41.875	0.6915
502848	8518	44.553	0.7042

**Table 2:** Video parameters for Little’s theorem.

Arrival time	Departure time	Buffer length
(in seconds)	(in seconds)	(in bits)
0.1105	24.7296	283554
24.8414	46.3429	238058
46.4483	62.7653	177714
62.8706	86.1773	258482
86.2906	112.6488	293682
112.7647	143.5812	344650
143.6971	178.7735	394082
178.8831	213.4765	388290
213.5924	246.6938	371282
246.8058	276.2671	329146

**Table 3:** Audio parameters for Little’s theorem.

Arrival time	Departure time	Buffer length
(in seconds)	(in seconds)	(in bits)
0.7096	0.7184	5525
0.7185	0.7265	5982
0.7266	0.7322	6658
0.7373	0.7373	6276
0.7374	0.7455	6760
0.7456	0.7535	6334
0.7536	0.7617	4973
0.7618	0.7698	2628
0.7699	0.8579	4120
0.8580	0.8647	6344

## Results

The mean transmission time $\overline{x}$ for varying buffer lengths was calculated using the formula in (2) where $n$ is the number of measurements and $x_i$ is the transmission time for the $i$-th measurement. The standard deviation $\sigma$ measures the dispersion of transmission times around the mean and is computed using (3). The Pearson correlation coefficient $r$ quantifies the linear relationship between buffer length $L$ and transmission time $T$, given in (4).

2 $$\begin{aligned} & \overline{x} = {\frac{1}{n} \sum _{i=i}^{n} x_{i} } \end{aligned}$$

3 $$\begin{aligned} & \sigma = \sqrt{\frac{1}{N-1} \sum _{i=1}^N (x_i - \overline{x})^2} \end{aligned}$$

4 $$\begin{aligned} & r = \frac{n \sum (LT) - \sum L \sum T}{\sqrt{\left(n \sum L^2 - (\sum L)^2\right)\left(n \sum T^2 - (\sum T)^2\right)}} \end{aligned}$$

where: n = number of data points, *L* = buffer length, *T* = transmission time, $\sum X = \textit{sum of all values in } X,$ $\sum Y = \textit{sum of all values in } Y$, $\sum XY = \textit{sum of the product of corresponding values in } X \& Y$, $\sum X^2 = \textit{sum of the squares of values in } X$, $\sum Y^2 = \textit{sum of the squares of values in } Y$.

 By taking the transmission time of Video as X and the transmission time of audio as Y the result values have been tabulated in Table 4.

**Table 4:** Statistical analysis.

Method	Value	
	Video	Audio
Mean transmission time	28.64081 s	0.53048 s
Standard deviation of transmission time	10.34539 s	0.15930 s
Pearson correlation coefficient	0.99999	0.09620

Little’s Theorem is a fundamental result in queuing theory that relates the average number of items in a stationary system (*L*), the average time each item spends in the system (*W*), and the average arrival rate of items ($\lambda$). It can be expressed mathematically as:

5 $$\begin{aligned} L = \lambda \times W \end{aligned}$$

where: *L* is the average number of items in the system, $\lambda$ is the average arrival rate of items, *W* is the average time each item spends in the system.

### Applications in VLC system

1. *System Stability* The stability of the VLC system can be determined using Little’s Law. If the average arrival rate ($\lambda$) is less than the average departure rate, the system is stable.
2. *Performance Evaluation* Little’s law can also be used for analyzing the system performance based on the representation of the average number of frames in the system and the average time for each frame to be spent in the system.
3. *Capacity Planning* Little’s Law helps in capacity planning by revealing what effect the changes in arrival rate or duration of the treatment have on the number of frames being accommodated in the system.

For the proposed system, the results using Little’s theorem in (5) obtained are tabulated in Table 5.

**Table 5:** Result of Little’s theorem.

Parameter	Value	
	Video	Audio
Total number of arrivals (N)	10	10
Total time (T)	276.1566 s	0.1551 s
Average arrival rate $(\lambda )$	0.03621 s	0.7518 s
Average time spent by a frame (W)	27.51515 s	0.0149 s
Average number of frames (L)	0.9963	0.9619

## Conclusion and future scope

In this section, we briefly conclude the work based on statistical analysis, Little’s theorem, and energy efficiency. We also highlight the limitations and possible future enhancements.

A. *Conclusion based on statistical analysis* the obtained video dataset exhibits a strong positive correlation between the buffer length and transmission time and also between buffer length and illumination levels. Higher illumination leads to longer transmission times showing the impact of environmental conditions on system behaviour. Whereas the audio transmission provides a high data rate compared to the video transmission. However, there is always the influence of external factors such as signal strength, latency, and noise in audio transmission performance. Further optimization strategies such as optimizing modulation techniques, reducing latency, and enhancing signal quality can optimize system performance and reduce transmission time.
B. *Conclusion based on Little’s theorem*
  1. *Stability* Since the average number of frames in the system is close to 1 for both audio and video, the system is stable and is not overwhelmed by incoming frames.
  2. *Efficiency* The average time spent by a frame in the system is found to be 27.5151 seconds for video, which is relatively high indicating potential bottlenecks or delays in processing frames. But for audio, it is found to be 0.0149 seconds indicating that the system demonstrates efficient processing capabilities in the case of audio.
  3. *Reliability* The low average time spent by a frame in the system for audio feed indicates that frames are processed quickly and efficiently, suggesting that the system can consistently transmit audio data without significant delays or interruptions.
C. *Energy Efficiency* The system uses solar panels for energy harvesting and battery charging. While the current configuration generates sufficient energy (0.6 Wh) to complement the battery, scaling up the panel dimensions significantly enhances energy availability, supporting high-power applications.

### Limitations of the work and possible future enhancements

1. *Limited VLC Range and Reliability* VLC requires both the receiver and the transmitter to be in the line of sight at all times which is difficult to maintain outdoors. Also, VLC communication may be limited in certain conditions like extreme weather areas with low visibility, impacting the navigation command transmission reliability. When VLC experiences significant attenuation from external factors, combining it with conventional radio frequency (RF) transmission can offer a reliable fallback strategy. This hybrid strategy guarantees constant communication. The effectiveness of VLC under challenging circumstances can be enhanced by using specific wavelengths of light that are less impacted by weather (such as near-infrared rather than visible light). High-sensitivity photodetectors’ superior capacity to detect blurred light signals increases communication reliability in low-visibility situations. Reliability can be improved by dynamically adjusting the system’s operating parameters (such as gearbox power and angle) and monitoring environmental variables in real time.
2. *Power Dependency on Solar Panels* Reliance on solar panels for power may limit operation in cloudy or low-light situations. To overcome this, during diffused sunlight, energy can be generated by installing concentrators, such as reflectors or lenses, around the panels to help focus more sunlight onto the panel surface. Thin-film solar panels can produce energy even with dispersed sunlight and typically outperform conventional silicon-based panels in overcast conditions. Install energy storage devices like sophisticated lead-acid or lithium-ion batteries. For usage in times of low light, these can store extra energy produced during the brightest parts of the day.
3. *Energy Optimization Challenges* Current solar panels have limited energy generation, future systems could integrate more efficient panels or adopt energy-efficient processing techniques to reduce overall consumption, ensuring better performance in high demand scenarios.
4. *Camouflage Challenges* While the robot has a camouflage feature, achieving complete invisibility in diverse terrains and lightning conditions is still challenging. To overcome this, flexible screens can give the robot the appearance of transparency by displaying colors or images dependent on camera inputs from its environment. We can use smart materials that adapt quickly to changing light circumstances by changing their optical characteristics (color, reflectance) in response to an electric voltage or light stimulus.
5. *Integration complexity* Combining camouflaging, VLC, audio/video streaming, navigation, and battery management into a single mobile system requires highly powerful hardware and sophisticated software integration. To overcome this, we must separate the program into discrete, independent modules that manage particular functions (such as communication, motion control, sensor data processing, and camouflage control). Each module must have a clear interface to communicate with other parts. Also, separate the hardware into distinct subsystems or parts that may be tested, replaced, or upgraded independently.

## Data Availability

The data will be made available on request. The request can be sent to the corresponding author.
